# Assessment of Dispersion and Bubble Entropy Measures for Enhancing Preterm Birth Prediction Based on Electrohysterographic Signals

**DOI:** 10.3390/s21186071

**Published:** 2021-09-10

**Authors:** Félix Nieto-del-Amor, Raja Beskhani, Yiyao Ye-Lin, Javier Garcia-Casado, Alba Diaz-Martinez, Rogelio Monfort-Ortiz, Vicente Jose Diago-Almela, Dongmei Hao, Gema Prats-Boluda

**Affiliations:** 1 Centro de Investigación e Innovación en Bioingeniería, Universitat Politècnica de València, 46022 Valencia, Spain; feniede@ci2b.upv.es (F.N.-d.-A.); rabes@ci2b.upv.es (R.B.); jgarciac@ci2b.upv.es (J.G.-C.); adiaz@ci2b.upv.es (A.D.-M.); gprats@ci2b.upv.es (G.P.-B.); 2 Servicio de Obstetricia, H.U.P. La Fe, 46026 Valencia, Spain; monfort_isaort@gva.es (R.M.-O.); diago_vicalm@gva.es (V.J.D.-A.); 3 Faculty of Environment and Life, Beijing University of Technology, Beijing International Science and Technology Cooperation Base for Intelligent Physiological Measurement and Clinical Transformation, Beijing 100124, China; haodongmei@bjut.edu.cn

**Keywords:** electrohysterography, uterine electromyogram, uterine electrical activity, preterm birth prediction, feature selection, genetic algorithm, bubble entropy, dispersion entropy, sample entropy, fuzzy entropy

## Abstract

One of the remaining challenges for the scientific-technical community is predicting preterm births, for which electrohysterography (EHG) has emerged as a highly sensitive prediction technique. Sample and fuzzy entropy have been used to characterize EHG signals, although they require optimizing many internal parameters. Both bubble entropy, which only requires one internal parameter, and dispersion entropy, which can detect any changes in frequency and amplitude, have been proposed to characterize biomedical signals. In this work, we attempted to determine the clinical value of these entropy measures for predicting preterm birth by analyzing their discriminatory capacity as an individual feature and their complementarity to other EHG characteristics by developing six prediction models using obstetrical data, linear and non-linear EHG features, and linear discriminant analysis using a genetic algorithm to select the features. Both dispersion and bubble entropy better discriminated between the preterm and term groups than sample, spectral, and fuzzy entropy. Entropy metrics provided complementary information to linear features, and indeed, the improvement in model performance by including other non-linear features was negligible. The best model performance obtained an F1-score of 90.1 ± 2% for testing the dataset. This model can easily be adapted to real-time applications, thereby contributing to the transferability of the EHG technique to clinical practice.

## 1. Introduction

Preterm birth (deliveries before 37 weeks of gestation [1]) affect more than 15 million persons worldwide, involving 5 to 18% of pregnancies [2]. It is one of the leading causes of infant mortality, varying from 90% (<28 weeks) to 10% as gestation time advances [2]. Two-thirds of preterm births happen after the spontaneous onset of labor, while the remainder are medically indicated because of maternal or fetal complications [3]. Being born too soon increases the risk of neurodevelopmental impairments and respiratory and gastrointestinal complications [3]. In the survivors, this has been associated with 20% mental retardation, 50% cerebral palsy, and 33% eye injuries [4]. Preterm births also have a serious economic impact on public health systems; the average cost of a preterm birth is 5–10 times higher than a term birth [5]. As for an extremely preterm baby, born before 28 weeks of gestation in Canada, it costs an average of $67,467 for the first ten years of its life [6]. Saving a baby weighing less than 750 g costs more than $117,000, the highest costing procedure in Canada’s public health system [7].

Several techniques have been proposed in the literature, such as monitoring uterine dynamics by tocodynamometry (TOCO), cervix length, Bishop score, and biochemical markers, to determine the risk of preterm birth [8]. Cervical length is seen as one of the best birth predicting methods [8], although several studies report that this criterion has proven to be insufficient or inaccurate [9,10]. Due to its low positive predictive values and sensitivities, routine cervical length assessment is not recommended in women at low risk of preterm birth [9]. Monitoring uterine activity is routinely used by obstetricians during labor. The two most widespread current methods are: directly through an intrauterine pressure catheter (IUPC) and indirectly through external TOCO. However, both have serious disadvantages and limitations in their use. IUPC is an invasive method that can only be used during labor, which can increase the risk of infection and may even harm the fetus or the mother. Although TOCO is safe, it uses pressure transducers on the abdomen and has poor sensitivity and precision [11]. Biological fluids have been used as biochemical markers to predict preterm births, although systematic reviews have indicated that no single biomarker or combination of such could be identified to reliably predict the preterm birth risk or pregnancy outcome [12]. None of them has been proven to objectively and precisely estimate the time of delivery and whether or not it will be premature [8]. Electrohysterography (EHG) has emerged as a promising technique to identify the risk of preterm birth due to its high sensitivity [13]. EHG is the recording of changes in bioelectrical potential of the uterine myometrial cells and can be picked up on the human abdominal surface. During pregnancy, the uterine myometrial cells undergo a process of increased excitability and bioelectric propagability due to the larger number of gap-junctions, which end up leading to coordinated high-intensity contractions that give rise to labor. These electrophysiological changes have been shown to be reflected in an increased EHG signal amplitude associated with the number of uterine cells involved in the contractions [13]. On the other hand, the shift of the EHG signal spectral content to higher frequencies has been associated with increased cell excitability [13,14]. The spectral content of the EHG signal has been widely studied and categorized by the frequency bandwidth: the whole bandwidth (WBW) ranges from 0.1–4 Hz, the slow wave, which is related to uterine contractions, and the fast wave which is usually subdivided into two components: fast wave low (0.13–0.26 Hz), which has been associated with signal propagation, and fast wave high (FWH) (0.34–0.88 Hz), which is related to cell excitability [13]. FWH is usually extended for study to 0.34–4 Hz [15].

Due to the non-linear nature of the biological system, other authors have proposed the use of different entropy measures to characterize EHG signals [16,17]. The approximate entropy algorithm aims at obtaining a statistically valid measure of entropy for noisy biomedical time series and represents the probability that similar patterns (delay vectors) in a time series will remain similar once the pattern lengths are increased (extended delay vectors), thereby providing a natural measure of the time series regularity [18]. Lemancevicz et al. found that approximate entropy computed in the 0.24–4 Hz bandwidth for women with threatened preterm birth who delivered in less than 7 days was significantly higher than those who finally delivered in more than 7 days, suggesting that the EHG signal becomes more irregular as pregnancy progresses [19]. However, approximate entropy has been shown to be a biased estimator and highly sensitive to the number of data samples [20], while sample entropy is a modification of approximate entropy, in which self-matches are not included in calculating the probability [21]. A lower value of sample entropy also indicates more self-similarity in the time series. Sample entropy is more independent of data samples and behaves more consistently than approximate entropy [21]. This latter has been widely used to characterize EHG signals for discriminating preterm and term birth records [15] and also for distinguishing between women with threatened preterm birth undergoing tocolytic therapy who finally deliver in less or more than 7 days [17,22]. In contrast to [19], the results of these works pointed to a reduction of entropy measurements (signal complexity) as labor approaches. Despite the promising results, sample entropy has been shown to have some drawbacks, e.g., it may be unstable and obtain unreliable results for short time series. It has also been shown to be sensitive to the configuration of its internal parameter values (embedding dimension m and scaling factor r) and can be too time-consuming for long data [23].

To deal with some of the deficiencies of sample entropy, fuzzy entropy is based on the concept of fuzzy sets. It presents a stronger relative consistency and shows less dependence on data length than sample entropy [23]. It has also been shown that the soft and continuous boundaries of fuzzy functions ensure continuity. Nevertheless, there are even more degrees of freedom for choosing internal parameters than for sample entropy, since both membership function and fuzzy power were introduced to define the boundary [24].

Since selecting the internal parameters is a critical issue in obtaining any entropy metrics, Manis et al. introduced a new definition of entropy known as *bubble entropy*, which is derived from permutation entropy, in which the vectors in the embedding space of the time series are ranked [25]. It quantifies the effort (number of swaps) required by the permutation process, which is carried out by the “bubble sort” algorithm, from which this measure obtains its name. By counting the number of swaps performed for each vector, a more coarse-grained distribution is obtained to compute the conditional Rényi entropy, which is the combination of the conditional permutation entropy [26] and Rényi permutation entropy [25,27]. Bubble entropy has been shown to be almost free of internal parameters, since the scaling factor r is totally eliminated in its definition, and the importance of embedding dimension m is significantly reduced. It has also been proven to be remarkably stable and has a greater power to distinguish subjects with congestive heart failure and normal sinus rhythm than both sample entropy and permutation entropy [25]. Manis et al. proposed using bubble entropy for discriminating congestive heart failure from a healthy control group [25].

In 2016, Rostaghi and Azami proposed using dispersion entropy, another entropy measure, to quantify the complexity of a time series [28]. Dispersion entropy is based on the symbolic dynamics or pattern and Shannon entropy to quantify the randomness of the times series. The concept of symbolic dynamic arises from a coarse-graining of the signal measurements, i.e., the signal is transformed into a new one with only a small number of patterns. The transformation is achieved by using mapping functions such as a linear or normal cumulative distribution function, among others [28,29]. In this way the study of the dynamic patterns of the time series is simplified to a distribution of symbol sequences. Dispersion entropy can detect a change of simultaneous frequency and amplitude and is relatively insensitive to noise since a small change in amplitude value will not vary the class label of the pattern [28]. Again, this entropy measure depends on the selection of the embedding dimension m and the number of classes c. For lower c values, there are few patterns to which to assign the time series, thus, underestimating the signal complexity, while if c is too high, small variations in the signal can cause a change of class, making it sensitive to noise [15]. It has been shown to perform better in detecting abrupt signal and noise robustness testing, has better stability for both simulated and real-word signals [30], and requires less computation time than sample entropy [29]. Dispersion entropy is also faster than other related entropy measures due to the fact that it does not need to either sort the amplitude values of each embedding vector or calculate every distance between any two composite delay vectors with embedding dimensions m and m + 1 [28]. Kafantaris et al. found that dispersion entropy obtained significantly higher values for both atrial premature beats and premature ventricular contraction electrocardiogram signals than healthy subjects’ [31]. Dispersion entropy of the discrete wavelet transformed electroencephalogram (EEG) has also been used for differential diagnosis of health control, mild cognitive impairment, and Alzheimer’s disease [32]. Tripathy et al. developed an automated sleep stage classification system, in which after some transformation of temporal EEG signals, they computed dispersion and bubble entropy. Their results could discriminate between different sleep stages with greater accuracy (>85%) [33].

To date, there has been no evidence of the utility of either bubble entropy or dispersion entropy for characterizing EHG signals; the aim of the present study was, therefore, to analyze the discriminatory capacity of bubble entropy and dispersion entropy EHG signals to differentiate between women who deliver at term and prematurely. We also attempted to determine whether these entropy measures can further improve the EHG feature space for predicting preterm birth by analyzing their complementary information to other EHG characteristics.

## 2. Materials and Methods

### 2.1. Database Description

**Figure 1:**
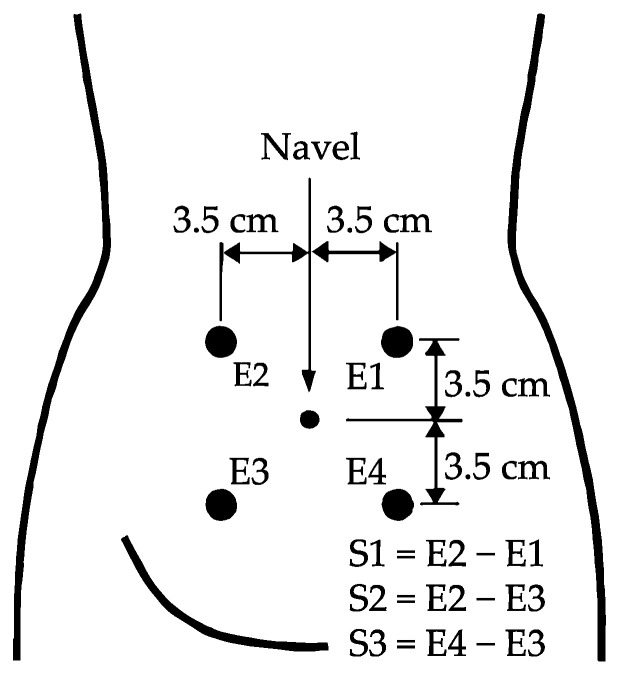
Recording protocol of EHG signals (modified from [34]).

A total of 326 EHG registers were analyzed from two public databases available in Physionet conducted on pregnant women between 22 and 37 weeks of gestation: “Term-Preterm EHG Database” (TPEHG DB) [15] and the “The Term-Preterm EHG Dataset with tocogram” (TPEHGT DS) [34] obtained by the Department of Obstetrics and Gynecology of the Ljubljana University Medical Center. Of the total, there were 275 term births and 51 preterm births (<37 weeks of gestations). The protocol used to obtain the EHG registers consisted of placing four electrodes (E1, E2, E3, and E4) on the woman’s abdomen to obtain three bipolar channels (S1, S2, and S3), with a pairwise distance of 7 cm (see Figure 1) [15]. The sampling rate was set to 20 Hz. The signals were further band-pass filtered between 0.1 and 4 Hz using a fifth-order digital zero-phase Butterworth filter.

### 2.2. EHG Signal Analysis

We first excluded from the study the corrupt signal segments from the recordings (motion-artifacts and respiratory interference) by a double-blind process conducted by two experts. A whole-window analysis was then carried out to characterize the EHG signals, since this has been shown to provide relevant information on the uterine electrophysiological state without the need to identify the EHG-bursts associated with contractions embedded in the records, which is more suitable for future “real-time” applications [15,16,17]. The analysis used 120 s moving windows with a 50% overlap, a good trade-off between information-loss and computational cost [22].

Since we aimed to determine whether the different entropy metrics could further enhance preterm birth prediction by analyzing their complementary information to other EHG characteristics, we computed a set of 46 temporal, spectral, and non-linear features [8] to characterize EHG signals, the analysis windows, and EHG recordings. These were organized in different feature groups, as shown in Table 1. We then computed the median value of the total analyzed windows to obtain a unique representative value for each recording session and channel. We calculated EHG signal peak-to-peak amplitude (App) in both the whole EHG (WBW) bandwidth 0.1–4 Hz and Fast Wave High (FWH) bandwidth 0.34–4 Hz since this latter seems to be more sensitive to labor proximity [15]. As the EHG signal is mainly distributed between 0.2 and 1 Hz, we computed various spectral features [16,17] to quantify the signal’s energy distribution, including dominant frequency DF1 and DF2 computed in the 0.2–1 Hz and 0.34–1 Hz ranges, respectively; mean frequency (MeanF) and power spectrum deciles (D1, …, D9) in the 0.2–1 Hz bandwidth; normalized subband energy (NormEn) (0.2–0.34 Hz, 0.34–0.6 Hz 0.6–1 Hz); high (0.34–1 Hz)-to-low (0.2–0.34 Hz) frequency energy ratio (H/L ratio); teager energy, and spectral moment ratio (SpMR). Since the analysis bandwidth is a key factor in estimating the non-linear features [16,17], we calculated them in both whole EHG and FWH bandwidths. These latter included: Lempel-Ziv index (binary (LZBin) and multi-state n = 6 (LZMulti) Lempel-Ziv index, which evaluates time series complexity by measuring how “diverse” the patterns embedded in a time series are; time reversibility (TimeRev), which estimates the dynamic flows’ similarity in forward (natural) time and reverse time and can be considered a measurement of the degree of signal nonlinearity [35]; Katz fractal dimension (KFD), as the uterine myoelectric activity has also been shown to possess fractal properties, which is another way of measuring self-similarity [36]; the Poincaré ellipse metrics were computed since the “present” EHG signal amplitude might significantly influence the “following” values. We, therefore, represented the Poincaré ellipse of consecutive EHG signal amplitudes (EHG[n] vs. EHG[n−1]) and extracted the main metrics (minor axis (SD1), major axis (SD2), square root of variance (SDRR, $\sqrt{{(\mathrm{SD}1}^{2}{+\mathrm{SD}2}^{2})/2}$), and SD1/SD2 ratio) [37]; spectral entropy (SpEn); sample entropy (SampEn); fuzzy entropy (FuzEn); dispersion entropy (DispEn), and bubble entropy (BubbEn). We performed an internal parameter sweep of the entropy measures to optimize their performance in discriminating preterm and term delivery by selecting the internal parameter combination associated with the lowest Wilcoxon Rank-Sum Test *p*-value when comparing preterm and term groups. For SampEn, the embedding dimension m was grid searched from 2 to 5, while the scaling factor r swept from 0.05 to 0.3 with a step of 0.05 times the standard deviation of the time series. Both the m and r ranges were considered to achieve reliable results for physiological data [38]. Due to the high number of degrees of freedom required for internal parameter selection to estimate fuzzy entropy (embedding dimension m, scaling factor r, fuzzy power n, and membership function), we evaluated several fuzzy membership functions as proposed in [24]: triangular, trapezoidal, Z-shaped, bell-shaped, gaussian, constant-gaussian, and exponential functions. For each membership function, we used the optimized parameter r and power n for discriminating biomedical signals [24], sweeping the embedding dimension from 2 to 5. We varied the dispersion entropy internal parameter m from 2 to 5 and the class number c between 3 and 9. The range for m and c was selected according to the literature, satisfying c^m^ < Z, Z being the length of the time series [29]. We assessed the performance of five mapping functions to compute DispEn: linear, normal cumulative distribution function, tangent sigmoid, logarithm sigmoid, and the sorting method. Finally, we varied the embedding dimension of bubble entropy m from 2 to 40, since stability has been shown to improve as m increases [25]. For SampEn, FuzEn, SpEn, and DisEn, the time delay parameter was fixed to 1 to avoid loss of information of high frequency components without excessively increasing the computational cost [24,28]. Table 2 shows the optimized internal parameters of the entropy measures best able to discriminate preterm and term records (lowest *p*-value of Wilcoxon Rank-Sum Test). Each optimized entropy was included for further analysis to predict preterm birth.

To analyze the clinical value of different entropy measures for predicting preterm birth, we designed prediction models 1 and 2 using a subset of entropy measures previously used in EHG analysis: En_SFS_ set (sample entropy, fuzzy, and spectral entropy) and all entropy measures En_ALL_ (see Table 3). We also defined models 3–6 to determine whether entropy measures provided complementary information to other EHG characteristics and obstetric data. Table 3 shows the input features of the six preterm birth prediction models developed in this work.

### 2.3. Classifier Design and Evaluation

Due to the fact that only about 12% of the women undergoing regular check-ups deliver prematurely, there is a high imbalance rate between the two target classes in the original database, term and preterm. So as to mitigate the bias of conventional classification algorithms towards the majority class, obtaining low sensitivity for true preterm birth, we used the synthetic minority oversampling technique (SMOTE, k = 5) to obtain balanced preterm and term birth data [39]. This latter consisted of generating synthetic samples for the minority class, taking into account the original feature space. The conventional holdout method (30 partitions) was used to design and validate the classifiers. For each partition, the whole balanced database was randomly split into training (1/3), validation (1/3), and testing (1/3) with the same proportion between classes for designing, validating, and testing the classifier. We used the same partitions for designing and testing the classifier to compare the performance of the different models (see Table 3) for predicting preterm birth.

Since we attempted to evaluate the complementary information between entropy metrics and linear and other non-linear EHG features, we preferred to use a feature selection technique rather than dimensionality reduction. Feature selection is the process of obtaining a subset of relevant features to construct a machine learning model, it removes “irrelevant” features that do not contribute much to the classification problem and keeps the most relevant and complementary information to discriminate the target classes. The computational cost is also reduced by removing some of the features [40]. To optimize feature subset selection, we used the genetic algorithm, which is a random search strategy that provides a trade-off between classification performance and search complexity for a moderate and/or large number of features [41]. Both population size and genome length were fixed to the number of the model’s input features (N) [42]. The tournament function was established with a size of 2 and an elite count of 2 to create the next generation population [42]. The crossover probability of combining the genetic information of parents to generate new offspring was typically assumed between 0.6 and 1, increasing the randomness of the children generation for a lower value [43]. The convergence to a lower minimum is better with low values (<0.1) of mutation probability, which is used to maintain the genetic diversity between generations [43,44]. Arithmetic crossover was used with a probability of 0.8 and uniform mutation with a probability of 0.01. Finally, the genetic algorithm’s termination condition was achieved if the fitness function did not improve noticeably for 150 consecutive generations (differential tolerance: 10^−6^).

To analyze the complementary information between input features we preferred to use linear classification methods without any complex data transformation by the linear discriminant classifier (LDA) to design the preterm birth prediction model.

**Figure 2:**
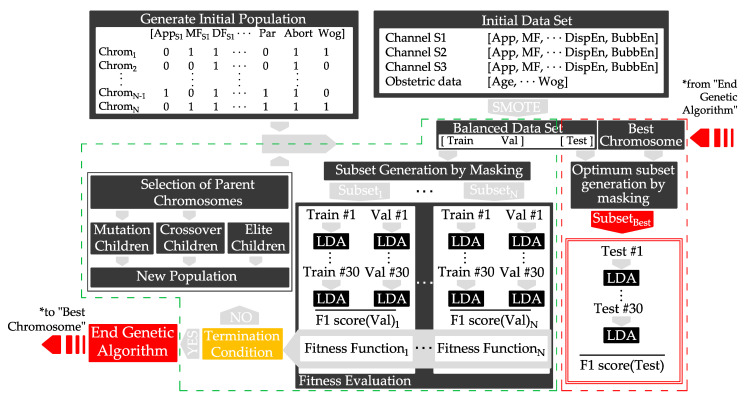
Diagram of the genetic algorithm for selecting the optimized feature subset to predict preterm birth based on EHG (green dashed line). The red dashed line represents the calculation of the performance of the test group masked by the best chromosome obtained from the optimization of the genetic algorithm, considering: training dataset (Train), validation dataset (Val), testing dataset (Test), chromosome (Chrom), population size (N).

Figure 2 shows a scheme of the optimization of features. Initially, we started balancing the original imbalanced dataset for each initial feature of the model (see Table 3). In the first step of the genetic algorithm, a set of randomly generated chromosomes masked the balanced data set, creating a feature subset. The mask (selected features), which corresponded to an i-chromosome, was set to the balanced data set obtaining the i-subset, with 1 ≤ i ≤ N, N being the model input features. We then used the LDA classifier to design the prediction model using each feature i-subset for the training dataset. The model was then scored by a fitness function in the validation dataset, defined as the mean F1-score of the 30 validation datasets weighted by the number of features being used in each iteration.

(1) $$\mathrm{Fitness}\;\mathrm{function}=\max\{\bar{F1-\mathrm{score}}\;\cdot \;(\mathrm{NFeat}\;-\;\mathrm{NCFeat})\}$$

where:

- NFeat is the number of features of the initial set.
- NCFeat is the number of features of the current subset.

The two best scored chromosomes (elite children) and new ones derived from mutation and crossover processes created a new population. This process was repeated until the termination condition was achieved, giving rise to the optimum feature subset (best chromosome).

For the different performance comparisons of the prediction models, we also computed the following metrics for the dataset testing: F1-score, accuracy, sensitivity, specificity, positive predictive value (PPV), negative predictive value (NPV), and area under curve (AUC). Likewise, we carried out the Friedman nonparametric test to analyze statistical differences in different metrics for the different models. The Wilcoxon Rank-Sum test was then used for pair-wise evaluation of the classifiers, checking the similarity of their performance.

## 3. Results

**Figure 3:**
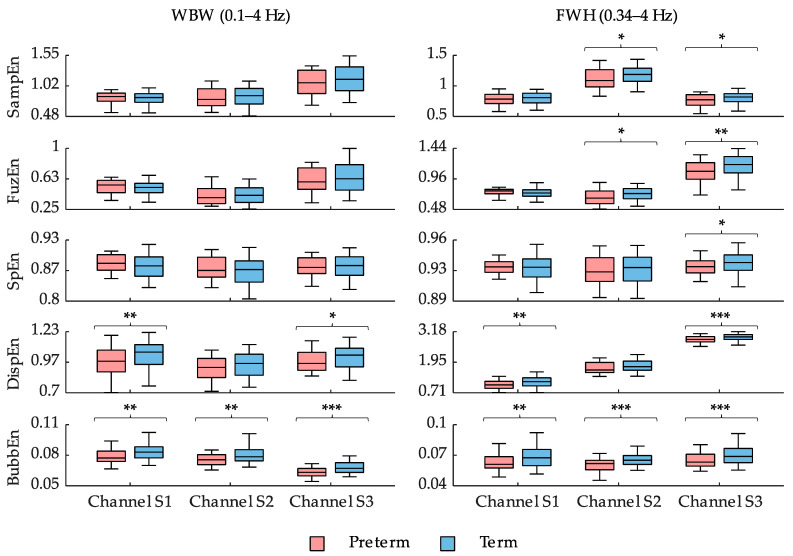
Box and whisker plot distributions of SampEn, FuzEn, SpEn, DispEn, and BubbEn using the optimal configuration of internal parameters indicated in Table 2 computed from EHG signals in different bandwidths and channels. *, ** and *** mean significant statistical difference (*p*-value ≤ 0.05, ≤ 0.01, and ≤ 0.001, respectively) between preterm and term records.

Figure 3 shows box and whisker plots of different entropy measures using the optimal configuration of their internal parameters for both the whole and FWH bandwidths for preterm and term birth records. In general, the different entropy measures from the preterm group showed lower values than those from the term group, suggesting increased signal predictability as labor approaches, although some controversial results were obtained with contradictory tendencies for sample, fuzzy, and spectral entropy in channel S1. Regardless of the recording channel, the entropy measures computed from the FWH bandwidth offered better separability between preterm and term groups and obtained lower *p*-values. The spectral entropy of the preterm group in the FWH bandwidth was significantly lower than that of the term group for channel S3. In sample and fuzzy entropy, statistically significant differences were found between the preterm and term groups for both channel S2 and S3. Fuzzy entropy seemed to offer slightly better separability for channel S3 when compared with sample entropy. Dispersion entropy obtained significantly different values in distinguishing preterm and term records for both channel S1 and S3, the latter obtaining the greatest separability. Finally, bubble entropy obtained significantly lower values for the preterm group than the term group for all the recording channels and the two bandwidths studied. Again, channel S3 obtained the best differentiation outcome between the preterm and term groups.

Table 4 shows the optimized feature subsets obtained for each of the feature models. SpEn was selected to consider only classical entropy metrics (En_SFS_). When extending the entropy characteristics with DispEn and BubbEn (En_ALL_), only the latter was selected. In the rest of the models some features were shared between the optimum feature subsets. A large number of input features were computed for each model in different channels and EHG bandwidths. Due to the complexity of the multidimensional feature space and redundancy or complementarity between features, the genetic algorithm may reach the best chromosome with different combination of features for each model [44]. In spite of these issues, many features are part of the best chromosome of the models developed. For linear metrics, DF1, NormEn in 0.1–0.34 Hz, D6, D8, and SpMR were selected for every optimum subset. KFD in the whole bandwidth was selected in the two models that included it as input features, i.e., LNL and LNLEn_ALL_. Regarding entropy metrics, only bubble entropy appeared in every optimum subset. Only week of gestation was chosen in all cases for obstetric features. Features such as Lempel-Ziv multistate, time reversibility, or Poincaré ellipse metrics were never selected from any channel or bandwidth, which can be attributed to the fact that they contain redundant information with linear and entropy metrics.

**Figure 4:**
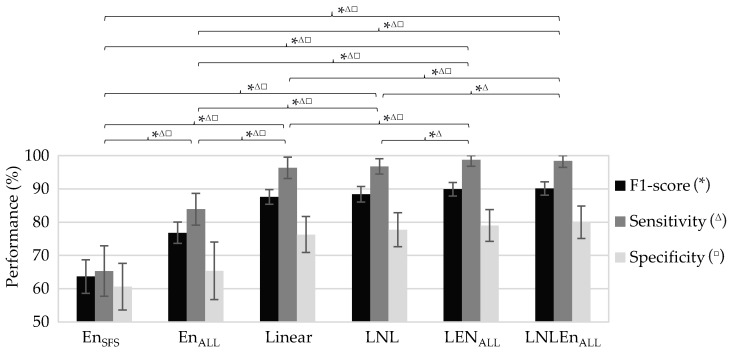
Comparison of the metrics’ performance in the testing dataset for different preterm birth prediction models *, ^Δ^, and ^□^ mean a significant statistical difference (*p*-value ≤ 0.05) between classifiers’ performance in F1-score, sensitivity, and specificity, respectively, by the Wilcoxon Rank-Sum test.

**Figure 5:**
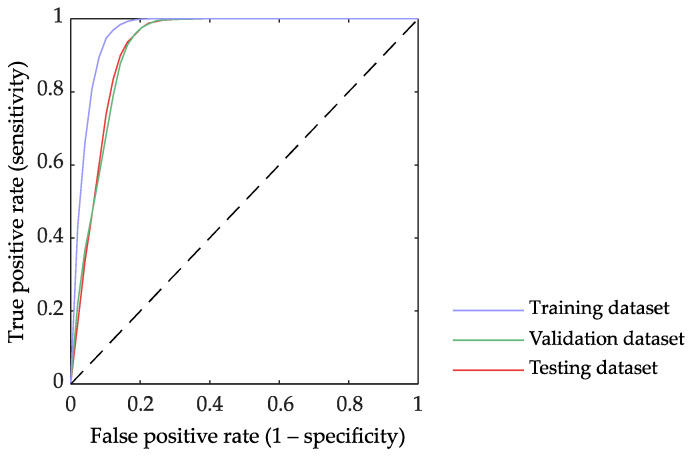
ROC curves of the LNLEn_ALL_ model that used linear, all non-linear EHG features and obstetric data including optimized feature subset for the training, validation, and testing dataset.

As expected, in the preterm birth prediction models, the training dataset always obtained higher performance than the validation and testing datasets, the performance of these two latter being similar. Since the testing dataset performance denotes the model generalization capacity for the “never seen” incoming data, we only show in Table 5 the average performance of the testing dataset for the different preterm birth prediction models. Figure 4 shows the outcome of pairwise comparisons of the performance metrics of these prediction models using the Wilcoxon Rank-Sum test. Moderate average performance with relatively high variability was achieved when using only entropy measures for predicting preterm birth, the average F1-score being 63.7 ± 5.1% and 76.8 ± 3.2% for En_SFS_ and En_ALL_, respectively. The inclusion of dispersion and bubble entropy significantly enhanced the prediction model performance, and only BubbEn_WBW, S2_, BubbEn_WBW, S3_ are part of the En_ALL_ best chromosome. The Linear model, which used linear EHG features and obstetric data, provided a significantly higher F1-score (87.6 ± 2.2%) than En_SFS_ and En_ALL_. The inclusion of other non-linear features (LNL model with binary and multistate Lempel-Ziv, Time reversibility, Katz fractal dimension, and Poincaré ellipse metrics) only provided a slightly better prediction outcome than Linear, without any significant difference (Linear 87.6 ± 2.2% vs. LNL 88.4 ± 2.3%), while entropy measures seemed to complement linear EHG features, obtaining a higher performance (Linear 87.6 ± 2.2% vs. LEn_ALL_ 89.9 ± 2%) with a statistically significant improvement in sensitivity, specificity, and F1-score. The best performance was achieved by the LNLEn_ALL_, which used both linear and all non-linear EHG features, including all the entropy measures, with an average F1-score of 90.1 ± 2%. The LNLEn_ALL_ metrics were significantly higher than those of the other models (see Figure 4), except for LEn_ALL_, for which no significant difference was found in any metric. The variability of LNLEn_ALL_ metrics between partitions, especially for sensitivity, was lower than other models. Figure 5 shows the ROC curve of LNLEn_ALL_ for training, validation, and testing a dataset. The ROC curve shows how the training partition presents the best performance with a larger area under the curve. The validation and testing partitions obtained a lower area under the curve than the training partition and an almost overlapping curve, which suggests a high power of generalization and, therefore, minimizes bias and variance in the classification.

## 4. Discussion

In this work, we compared five entropy measures from three EHG channels computed in both FWH and WBW bandwidths for distinguishing between preterm and term delivery records. Our results showed that the EHG metrics from channel S3 generally obtained lower *p*-values between preterm and term delivery metrics, suggesting greater class separability. This finding is consistent with those found by other authors, who attempted to predict preterm birth using information extracted from the S3 channel due to its higher signal-to-noise ratio [45,46,47,48]. We also confirmed that the different entropy measures computed in the FWH bandwidth provided higher separability between preterm and term delivery records than the WBW bandwidth, as we found in a previous work [16]. As for entropy measures, sample entropy was widely used for characterizing EHG signals acquired in women who had had regular check-ups, women with threatened preterm birth, and those who underwent labor induction [8,22,49]. Both fuzzy entropy and spectral entropy were previously proposed to distinguish preterm and term records [17]. As far as we know, this is the first time EHG has been characterized using dispersion entropy and bubble entropy, which have also been used for quantifying the regularity of other biomedical signals [25,31,32,33].

With regard to the application of dispersion and bubble entropy to EHG characterization, despite eliminating the scale factor r in bubble entropy that makes it easier to search for optimization parameters, it still presents a certain dependency on embedding dimension m. In our application, preterm records obtained lower bubble entropy values than term records for high embedding dimension m, suggesting increasing signal predictability as labor approaches [8,13]. In contrast, a low embedding dimension may lead to physiological misinterpretation throughout pregnancy. This finding was consistent with the observation made by Manis et al., with respect to the increase in the stability of the entropy measure as the embedding dimension increases [25]. We also found bubble entropy to be less sensitive to the signal bandwidth considered in the computation. In contrast, dispersion entropy was more sensitive to the signal bandwidth in which we computed this measure (see Figure 3, WBW vs. FWH). This may be due to the fact that dispersion entropy not only detects the signal complexity, but also instantaneous amplitude and frequency fluctuations [29].

We found that bubble entropy outperformed dispersion entropy and fuzzy entropy in discriminating preterm and term delivery patients and that these latter outperformed sample and spectral entropy. Our results agree with those of other authors who stated that dispersion entropy was found to be more consistent than sample entropy in characterizing the effect of age on the intrinsic stride-to-stride dynamics for gait maturation evaluation and in discriminating the non-invasive blood pressure signals of Dahl salt-sensitive hypertensive rats and rats protected from high-salt-induced hypertension [29]. Azami et al. also showed that dispersion entropy outperformed both sample entropy and fuzzy entropy for characterizing resting-state magnetoencephalogram regularity in Alzheimer’s disease [50]. Fuzzy entropy is more effective than sample entropy and approximate entropy for distinguishing Alzheimer patients from normal subjects [51].

We also attempted to determine the redundancy and complementary information between input features, since redundant and/or irrelevant features may lead to high computational complexity and overfitting problems, thereby increasing the variance of the prediction model without reducing its bias [52]. Information redundancy can be detected by analyzing mutual information in a multidimensional feature space to obtain a high correlation between the chosen features subsets and the target class [53]. Nevertheless, estimating the mutual information (especially through estimating probability density functions) between high-dimensional variables is a hard task in practice due to the limited number of available data points for real-world problems [52]. In this work, we used a wrapper method based on a genetic algorithm for selecting complementary features to enhance the prediction model outcome while keeping redundancy features out. This latter has also been proven to outperform the filter method for predicting pregnancy and labor contractions [54].

While previous studies reported that non-linear and entropy features have been shown to better characterize the EHG signal than linear metrics [55,56], our results showed that linear, non-linear, and entropy metrics complement each other for differentiating between preterm and term deliveries. This agrees with those of other authors who proposed using sample entropy together with linear features (root mean square, peak frequency, and median frequency) and obstetrical data for distinguishing term and preterm delivery records and achieved an AUC of 95% using a cross validation technique [45]. In a later work, feature ranking was proposed to determine the optimized feature subset, achieving a similar AUC of 94% using sample entropy, log detector, and other linear metrics as input features [47]. We found a great deal of redundant information between non-linear features, and only 4 of 78 non-linear and entropy features were included in the optimized feature subset for LNLEn_ALL_. This could be associated with the fact that these latter attempted to quantify the same phenomena: signal regularity and complexity. Only entropy measures complement linear features, obtaining a significantly higher prediction performance (see Table 5 Linear vs. LEn_ALL_); the improvement of prediction performance when including other non-linear features being negligible (Linear vs. LNL). For entropy measures, fuzzy and bubble entropy offered complementary information for predicting preterm and term delivery records, which were included in the optimized feature subset (see Table 4). Spectral, sample, and dispersion entropy were more likely to be redundant than fuzzy and bubble entropy. In our previous work [57], a similar input feature to the one included in this work (adding, in this case, dispersion and bubble entropy) was optimized using a genetic algorithm for feature selection and LDA for classifying. The results revealed that selected linear features maintain a correlation with those in this work. Dominant frequency in 0.1–1 Hz and 0.34–1 Hz, normalized energy in 0.1–0.34 Hz and spectral moment ratio were chosen in both studies. Decile 5 seemed to be a good discriminative feature, but in this case, it seems to be replaced by deciles 6, 8, and 9, indicating their complementarity and redundancy with decile 5. In contrast, peak-to-peak amplitude was selected by the genetic algorithm in other computed bandwidths not added to the input feature of the present work. In the non-linear parameters, only the Katz fractal dimension in the whole bandwidth appeared in the optimum feature subset in LNL and LNLE_nALL_. For entropy measures, the double selection of bubble entropy and the lack of other non-linear and entropy metrics in the final subset suggest that bubble entropy has a high discriminating power between term and preterm cases. As bubble entropy keeps enough redundant and complementary information with non-linear and other entropy metrics, they were not used in the optimum feature subset. Our results agree with those of Cuesta-Frau, who suggested that bubble entropy may offer complementary information to other entropy measures, such as permutation entropy, for predicting the risk of developing diabetes [58]. In this regard, fuzzy entropy was also found to complement entropy measures as the distribution entropy for differentiating both ictal and interictal EEG from normal EEG and for discriminating ictal from interictal EEG [59].

Our results are hardly comparable to many previous works in preterm birth prediction systems that used cross validation methods to design and validate the classifiers, without determining the real generalization capacity for incoming “never seen” data by the classifiers [45,46,47,60]. The results of the prediction model obtained in this work even outperformed the results of our previous work, in which principal component analysis was used for dimension reduction of input features and multilayer perceptron artificial neural network to implement the classifier [61]. We believe that this prediction performance improvement was mainly due to the information optimization in feature space using the genetic algorithm, which can eliminate any redundant information and irrelevant features while keeping in the complementary information [41]. In addition, by optimizing information in feature space, we showed the feasibility of designing a preterm birth prediction system using simple linear classifiers, which are easily interpretable by clinicians. The complex classification algorithms, which can only be interpreted by experts, such as artificial neural network and/or support vector machines, can be dispensed with, which will considerably improve the transferability of the technique to clinical practice [62]. Instead of the mean efficiency index, which has been proposed as a robust indicator of uterine electrical activity efficiency from multichannel recordings [61], in this work, we used the EHG features extracted from the three individual channels since there is a greater degree of freedom in combining the information extracted from them.

Ahmed el al. used multivariate multiscale sample and fuzzy entropy in addition to univariate metrics to capture cross-channel dynamics of multichannel EHG recording and to characterize the interaction between the variates of complex systems to successfully discriminate between women who finally delivered at term and those who did so prematurely [63]. We found that the multivariate sample, fuzzy, and dispersion entropy measures obtained a relatively low model performance (~60%) for the test dataset (result not shown here for the sake of brevity). The addition of these multivariate entropy measures to the univariate measures did not significantly improve the model performance, which means that no additional relevant information can be obtained from them.

In spite of its promising results, the present study has certain limitations that should be pointed out. There are various factors that make the transfer of the EHG technique to clinical practice difficult; the databases are small and highly imbalanced for the preterm birth class; for instance, in the public databases used in this work, the term/preterm ratio is around 7 to 1. A larger database will be needed to assess the robustness of these preterm birth prediction systems if they are to be used in clinical practice. Second, the lack of a standard protocol for the electrode position in EHG recordings is another factor that makes a shared database difficult. On the other hand, most of the prediction systems are based on neural networks or support vector machine, multilayer perceptron, or similar algorithms, which involve non-linear transformations of the input EHG features into high dimension space, in which data from the target classes offer better linear separability [40]. This could give rise to good prediction performance even when the input features apparently do not contain individually information to differentiate the target classes. Obstetricians often consider this type of classification algorithm as a “black box” or a “mathematician’s gadget” due to its being difficult to interpret [62] and so find it difficult to trust the predictions of these complex classifiers. In a previous study, we, therefore, attempted to develop a preterm birth prediction model using simple classifiers to avoid complex artificial intelligence algorithms, whose success depends mainly on the information embedded in the features [57]. Other clinically relevant measures, such as cervical length, fetal fibronectine, and/or interleukin 6, which has been proven to be one of the more effective techniques to predict preterm birth [8], were missing in these databases [15,34]. This could partly be due to the fact that EHG signals were recorded from regular check-ups in women that did not show symptoms of preterm labor risk, and these measurements are not usually performed in this scenario. The inclusion of these additional clinical data to the predictor model could, therefore, further improve preterm birth prediction performance [17]. The commonly-used SMOTE oversampling technique was employed to mitigate the imbalanced class problem [39]. Future studies should focus on the design and validation of preterm birth prediction systems using specifically imbalanced data learning algorithms.

## 5. Conclusions

Both dispersion and bubble entropy can be used to characterize EHG signals, providing a higher between-class distance for distinguishing between preterm and term delivery records than sample, fuzzy, or spectral entropy. A feature-selection method based on a genetic algorithm was used to determine redundant and complementary information between linear and non-linear EHG features. We found that non-linear features contained a great deal of redundant information, as did the different entropy measures. Nevertheless, the entropy measures offered complementary information to linear features and could achieve a significantly higher performance for predicting preterm birth. Bubble entropy was declared to be a high-performance term-preterm discriminator, even improving on dispersion entropy in individual and multidimensional approaches. By optimizing the information in the feature space using the genetic algorithm, we were able to design a preterm birth prediction system using a simple linear classifier that yielded an average F1-score of 90.1 ± 2% for the test dataset. These results suggest that the proposed system has a high generalization capability for “never seen” incoming data and has great potential to bring the EHG technique closer to clinical practice.

## Figures and Tables

**Table 1:** EHG features for predicting preterm birth including the composition of each feature group. SampEn, FuzEn, and SpEn were included in En_SFS_ and En_ALL_ subsets to analyze the additional value of bubble entropy and dispersion entropy for predicting preterm birth in relation to other entropy measures.

	**Linear Features (L)**	Non-Linear Features (NL)	En_SFS_	En_ALL_	Obstetric Data
Number of features	20/channel	16/channel	6/channel	10/channel	5
Included features	App MeanF. DF1, DF2 NormEn H/L Ratio [D1−D9] Teager Energy SpecMR	LZBin LZMulti (n = 6) TimeRev KFD SD1 SD2 SDRR SD1/SD2	SampEn FuzEn SpEn	SampEn FuzEn SpEn DispEn BubbEn	Maternal age Parity Abortions Weight Week of gestation at recording time (Wog)

**Table 2:** Optimum internal parameters of each entropy measure in 0.1–4 Hz and 0.34–4 Hz bandwidths for channels S1, S2, and S3.

	Channel S1	Channel S2	Channel S3
SampEn_WBW_	m = 3, r = 0.15	m = 3, r = 0.1	m = 2, r = 0.1
SampEn_FWH_	m = 2, r = 0.3	m = 3, r = 0.1	m = 2, r = 0.3
FuzEn_WBW_	m = 5, r = 0.0077, n = 3, exponential function	m = 5, r = 0.0077, n = 3, exponential function	m = 2, r = 0.0077, n = 3, exponential function
FuzEn_FWH_	m = 5, r = 0.0077, n = 3, exponential function	m = 5, r = 0.0077, n = 3, exponential function	m = 2, r = 0.0077, n = 3, exponential function
DispEn_WBW_	m = 2, c = 3, linear	m = 2, c = 3, linear	m = 2, c = 3, linear
DispEn_FWH_	m = 2, c = 3, linear	m = 3, c = 4, linear	m = 2, c = 7, logsig
BubbEn_WBW_	m = 23	m = 23	m = 26
BubbEn_FWH_	m = 25	m = 24	m = 24

**Table 3:** Composition of feature model depending on feature group considered.

Model	Acronym	Input EHG Features	Obstetrical Data	Initial Features
1	En_SFS_	En_SFS_	No	18
2	En_ALL_	En_ALL_	No	30
3	Linear	Linear	Yes	65
4	LNL	Linear, NL	Yes	113
5	LEn_ALL_	Linear, En_ALL_	Yes	95
6	LNLEn_ALL_	Linear, NL, En_ALL_	Yes	143

**Table 4:** Optimum feature subset reached for the different initial feature models, defined in Table 3. The number of features obtained in each model feature repeated in the best chromosome of all the classifiers that use them as inputs are marked in bold.

Input Features Acronym	Selected Feature Subset	N° of Features
En_SFS_	SpEn_WBW, S2_, SpEn_WBW, S3_	2
En_ALL_	BubbEn_WBW, S2_, BubbEn_WBW, S3_	2
Linear	App_WBW, S2_, DF1_S2_, **NormEn_0.2–0.34Hz, S2_**, NormEn_0.2–0.34Hz, S3_, H/L ratio_S1_, D3_S1_, **D6_S2_**, **D8_S3_, SpMR_S3_**, **WoG**	10
LNL	**DF1_S2_**, DF1_S3_, **NormEn_0.2–0.34Hz, S2_**, NormEn_0.2–0.34Hz, S3_, H/L ratio_S1_, D3_S1_, **D6_S2_**, **D8_S2_**, D8_S3_, D9_S2_, **SpMR_S3_**, LZBin_WBW, S3_, **KFD_WBW, S1_**, **WoG**	14
LEn_ALL_	App_FWH, S2_, **DF1_S3_**, DF2_S1_, **NormEn_0.2–0.34Hz, S2_**, NormEn_0.2–0.34Hz, S3_, **D6_S2_**, **D8_S3_**, **SpMR_S3_**, **BubbEn_FWH, S3_**, Abortions, **WoG**	11
LNLEn_ALL_	**DF1_S2_**, DF2_S1_, **NormEn_0.2–0.34Hz_**_, S2_, D3 _S1_, **D6_S2_**, **D8_S2_**, D9_S2_, **SpMR**_S3_, **KFD_WBW, S1_**, FuzEn_FWH, S1_**,** BubbEn_WBW, S2_, **BubbEn_FWH, S3_**_,_ **WoG**	12

**Table 5:** Average performance on testing dataset for the different feature subset models.

Input Features Acronym	F1-Score (%)	Accuracy (%)	Sensitivity (%)	Specificity (%)	PPV (%)	NPV (%)	AUC (%)
En_SFS_	63.7 ± 5.1	63 ± 4.6	65.3 ± 7.6	60.6 ± 7	62.5 ± 4.6	63.8 ± 5.4	66.3 ± 4.71
En_ALL_	76.8 ± 3.2	74.6 ± 4.2	83.9 ± 4.8	65.4 ± 8.7	71.1 ± 5	80.4 ± 4.5	80.8 ± 4.76
Linear	87.6 ± 2.2	86.3 ± 2.6	96.4 ± 3.2	76.3 ± 5.4	80.4 ± 3.5	95.6 ± 3.6	90 ± 2.5
LNL	88.4 ± 2.3	87.3 ± 2.7	96.8 ± 2.3	77.7 ± 5.1	81.4 ± 3.5	96.1 ± 2.6	91.7 ± 2.6
LEn_ALL_	89.9 ± 2	88.9 ± 2.4	98.7 ± 1.9	79 ± 4.8	82.6 ± 3.3	98.5 ± 2.3	91.6 ± 2.8
LNLEn_ALL_	90.1 ± 2	89.2 ± 2.4	98.4 ± 1.9	79.9 ± 4.9	83.2 ± 3.3	98.2 ± 2.2	93.6 ± 2.3

## Data Availability

Access and download of the data used are openly available at https://physionet.org, accessed on 19 August 2021. “Term-Preterm EHG Database” (TPEHG DB) is available at https://www.physionet.org/content/tpehgdb/1.0.1/ (accessed on 9 September 2021) and the “The Term-Preterm EHG Dataset with tocogram” is available at https://physionet.org/content/tpehgt/1.0.0/ (accessed on 9 September 2021).
